# Effect of *Ku80* Deficiency on Mutation Frequencies and Spectra at a LacZ Reporter Locus in Mouse Tissues and Cells

**DOI:** 10.1371/journal.pone.0003458

**Published:** 2008-10-20

**Authors:** Rita A. Busuttil, Denise P. Muñoz, Ana Maria Garcia, Francis Rodier, Woo Ho Kim, Yousin Suh, Paul Hasty, Judith Campisi, Jan Vijg

**Affiliations:** 1 Buck Institute for Age Research, Novato, California, United States of America; 2 Department of Biology, University of Texas at San Antonio, San Antonio, Texas, United States of America; 3 Life Sciences Division, Lawrence Berkeley National Laboratory, Berkeley, California, United States of America; 4 Department of Pathology, Seoul National University College of Medicine, Seoul, Korea; 5 Department of Medicine, Albert Einstein College of Medicine, Bronx, New York, United States of America; 6 Department of Molecular Medicine, University of Texas Health Science Center, San Antonio, Texas, United States of America; University of Texas Southwestern Medical Center, United States of America

## Abstract

Non-homologous end joining (NHEJ) is thought to be an important mechanism for preventing the adverse effects of DNA double strand breaks (DSBs) and its absence has been associated with premature aging. To investigate the effect of inactivated NHEJ on spontaneous mutation frequencies and spectra *in vivo* and in cultured cells, we crossed a *Ku80*-deficient mouse with mice harboring a lacZ-plasmid-based mutation reporter. We analyzed various organs and tissues, as well as cultured embryonic fibroblasts, for mutations at the lacZ locus. When comparing mutant with wild-type mice, we observed a significantly higher number of genome rearrangements in liver and spleen and a significantly lower number of point mutations in liver and brain. The reduced point mutation frequency was not due to a decrease in small deletion mutations thought to be a hallmark of NHEJ, but could be a consequence of increased cellular responses to unrepaired DSBs. Indeed, we found a substantial increase in persistent 53BP1 and γH2AX DNA damage foci in *Ku80* ^−/−^ as compared to wild-type liver. Treatment of cultured *Ku80*-deficient or wild-type embryonic fibroblasts, either proliferating or quiescent, with hydrogen peroxide or bleomycin showed no differences in the number or type of induced genome rearrangements. However, after such treatment, *Ku80*-deficient cells did show an increased number of persistent DNA damage foci. These results indicate that *Ku80*-dependent repair of DNA damage is predominantly error-free with the effect of alternative more error-prone pathways creating genome rearrangements only detectable after extended periods of time, i.e., in young adult animals. The observed premature aging likely results from a combination of increased cellular senescence and an increased load of stable, genome rearrangements.

## Introduction

Ku80 is part of the Ku heterodimer, which acts with the DNA-dependent protein kinase (DNA-PK) catalytic subunit (CS) in the non-homologous end joining (NHEJ) pathway. This pathway joins the ends of DNA double-strand breaks (DSBs), whether spontaneously induced by genotoxic stress or generated by V(D)J recombination during the development of immunological diversity [1], [2]. NHEJ is critically important for repairing DSBs in adult mammals in which most cells are non-dividing and therefore unable to employ the alternative DNA DSB repair pathway, homologous recombination, which relies mainly on sister chromatids as templates for error-free repair [3]. DNA DSBs are highly toxic and need to be repaired swiftly to avoid DNA degradation and cell death, or genome rearrangements resulting from erroneous end joining. Ironically, both the presence and the absence of NHEJ are thought to be mutagenic, and the resulting mutations have been proposed to promote aging [4], [5].

During NHEJ, resection of the breaks before they can be re-ligated is thought to cause permanent loss of between 1 and 20 nucleotides. The continual loss of these small amounts of genomic sequence over the life time of an organism has been proposed to drive aging [4]. Alternatively, NHEJ is thought to prevent erroneous end joining leading to chromosomal translocations. Consistent with this view, *Ku80*-defective mice display multiple symptoms of premature aging and recent results indicate that DNA-PK_CS_ and Ku70-defective mice behave virtually identically [5], [6], [7]. Interestingly, while tumors in these mice appear earlier (as expected, since they live considerably shorter) there is no evidence for increased cancer, which is also true for other mice deficient in important genome maintenance pathways [8].

Little is known about the frequency, severity and types of DNA sequence changes that are associated with NHEJ or its absence in different mammalian organs and tissues. Although NHEJ is thought to cause small deletions surrounding the break, as mentioned above, there is ample evidence that DSBs can also be joined perfectly [9], [10]. Further, it has been suggested that at least two forms of NHEJ exist: one form in which DNA-PK_CS_ (generally restricted to vertebrates) plays a central role, and another (or more than one) alternative pathway in which end joining is significantly more error prone [11]. Alternative end joining pathways may be primarily responsible for joining breaks between different chromosomes, thereby causing translocations [12]. Chromosomal translocations, of course, are thought to be a major contributor to the development of cancer, but could also contribute to cell functional decline during aging [13].

Given that *Ku80* null mice are deficient in NHEJ, it is not clear why these animals show many prominent features of aging at an early age, yet do not have an increased cancer rate. To begin to understand this phenotype, we studied the mutational consequences of *Ku80* deficiency in the liver, spleen and brain of mice using a chromosomally integrated, concatamerized lacZ plasmid that is a sensitive reporter for both point mutations and large rearrangements [14]. We show that *Ku80*-deficiency results in an increased level of genome rearrangements, and a reduction in point mutations, in all three organs. In the liver, these mutational changes were accompanied by an increased number of cells harboring 53BP1/γH2AX foci, a hallmark of persistent DSBs and cause of cellular senescence. We suggest that premature aging in *Ku80*-deficient mice results from a combination of genome rearrangements and cellular senescence, the latter a consequence of chronic genotoxic stress that can also explain the reduced number of point mutations and reduced incidence of cancer.

## Results

### Spontaneous Mutant Frequencies *in Vivo*

**Figure 1:**
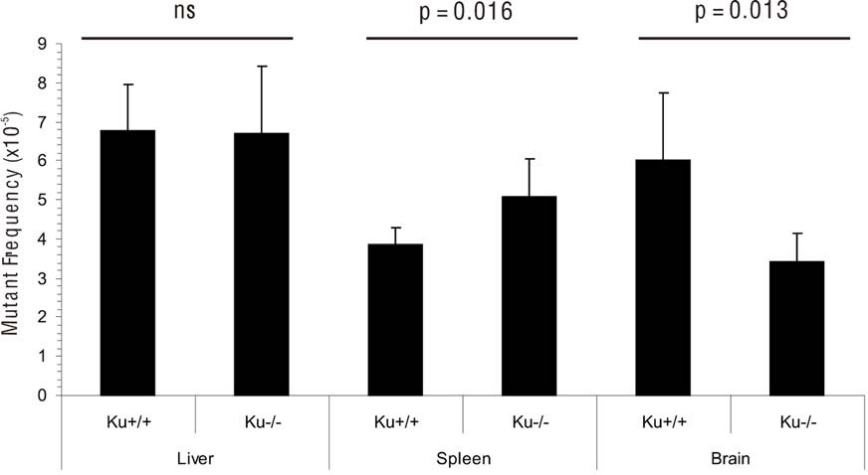
Spontaneous mutant frequencies in different organs from *Ku80* ^−/−^ and wt mice. Error bars indicate standard deviations (n = 5–8). Statistical tests were applied as described in the materials and methods to compare mutant frequencies between *Ku80* ^−/−^ and wt mice.

To assess spontaneous mutation frequencies in the *Ku80* ^−/−^ mice, we crossed them into mice harboring plasmids, integrated at chromosomes 3 and 4, containing the lacZ mutational reporter gene. These plasmids can be recovered from DNA extracted from various tissues and organs and lacZ inactivating mutations scored using a positive selection system (described in detail in [15]). We determined the spontaneous mutant frequencies for liver, spleen and brain from *Ku80* ^−/−^ and wild-type (wt) littermate control animals at 4–5 months of age (Fig. 1). Between 5 and 8 animals of each genotype were analyzed. Surprisingly, each tissue gave a different result with regard to the total mutant frequency. In the liver, we found no significant differences in mutant frequencies between wt and *Ku80* ^−/−^ animals (Fig. 1). In spleen, *Ku80* ^−/−^ mice had a significantly greater mutant frequency than wt mice (5.1×10^−5^ versus 3.8×10^−5^; Fig. 1, p = 0.016). In the brain, the mutant frequency of *Ku80* ^−/−^ mice was significantly lower compared to wt mice (3.4×10^−5^ versus 6×10^−5^; Fig. 1; p = 0.013). Hence, *Ku80* deficiency does not always lead to increased spontaneous mutation load *in vivo*.

**Figure 2:**
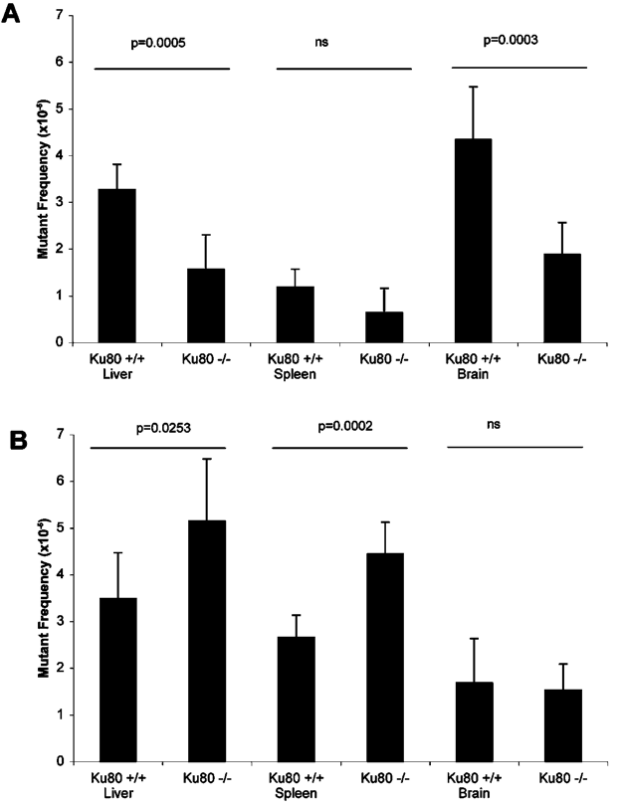
Frequencies of point mutations (A) and genome rearrangements (B) in liver, spleen and brain from *Ku80* ^−/−^ and wt mice, as determined from the mutants collected from the results shown in Fig. 1.

We next characterized the mutant lacZ genes in the livers, spleens and brains of *Ku80* ^−/−^ and wt mice. LacZ genes showing no change in size after restriction digestion (no-change mutants) are generally point mutations; lacZ genes showing a size-change after digestion (size-change mutants) are generally genome rearrangements with one break point within the lacZ gene and another elsewhere in the mouse genome [16]. Interestingly, although there was no statistically significant difference in the total mutant frequencies detected in the livers of wt and *Ku80* ^−/−^ mice, the ratio of genome rearrangements to point mutations differed significantly. In wt livers, the frequencies of no-change (3.3×10^−5^) and size-change mutations (3.5×10^−5^) were similar. However, in *Ku80* ^−/−^ livers, no-change (point) mutations were significantly reduced compared to wt livers (Fig. 2A; p = 0.0005) and this reduction was balanced by an increase in size-change (rearrangement type) mutations (Fig. 2B; p = 0.0253). *Ku80* ^−/−^ spleen also showed a decrease in the frequency of point mutations, but this decrease was not significant (Fig. 2A). However, as in the liver, *Ku80* ^−/−^ spleen showed a significant increase in the frequency of rearrangements relative to wt spleen (p = 0.0002; Fig. 2B). Finally, in the brain, the significant reduction in mutant frequency in *Ku80* ^−/−^ mice was due solely to a significant reduction in point mutations (Fig. 2A; p = 0.0003).

Together, these results indicate that *Ku80* deficiency leads to a significant increase in genome rearrangements, with a concomitant decrease in point mutations.

### NHEJ and Small Deletion Mutations

NHEJ is thought to be mutagenic because it often creates small deletions due to the necessary processing of DSB ends. We previously showed that small deletion mutations, i.e., affecting one or few basepairs, are not exceptional in brain of normal young mice [17]. To determine whether the reduced spontaneous mutant frequency we observed in the brains of *Ku80* ^−/−^ mice was due to preferential loss of small deletion mutations, we sequenced 11 lacZ mutants of the no-change class from wt and *Ku80* ^−/−^ mice. In Table 1 we subdivided all point mutations in basepair (bp) substitutions (mostly GC>AT transitions and GC>TA transversions) and deletions (of one or few bp). Comparison between wt and *Ku80* ^−/−^ mice indicated no evidence for a selective underrepresentation of small deletion mutations. Thus, NHEJ does not appear to be a major contributor to small deletion mutations in the mouse brain.

**Table 1:** Point mutations in brain of Ku80^−/−^ or wt mice.

Type of point mutation	Ku80^+/+^		Ku80^−/−^	
	n =	%	n =	%
Base pair substitutions	8	72.7	6	54.5
One or few base-pair deletions	2	18.2	5	45.5
One or few base-pair insertions	1	9.1	0	0.0

### Apoptosis, Cellular Senescence and DNA Damage Foci

We reasoned that the increased genotoxic stress resulting from the defect in DSB repair could readily increase DNA damage responses in tissues of *Ku80*-deficient mice. We tested this idea directly by analyzing liver, the most severely affected organ, for increased apoptosis or cellular senescence.

**Figure 3:**
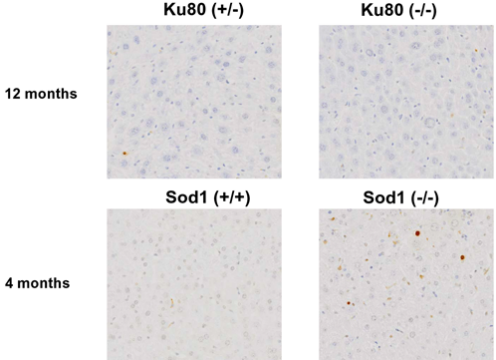
Increased apoptosis in liver of *Sod* ^−/−^ mice but not in *Ku80* ^−/−^ liver tissue. Representative images are shown from livers of *Ku80* ^−/−^ and wt mice at 12 months and *Sod1* ^−/−^ and wt mice at 4 months. Cells undergoing apoptosis were detected by *in situ* labeling of nuclear DNA fragmentation (TUNEL) as described in the text.

We examined apoptotic cell death using apoptosis-induced DNA fragmentation and *in situ* labeling (TUNEL) of the fragments in liver tissue of *Ku80* ^−/−^ mice of 10 weeks (n = 5), 6 months (n = 5) and 12 months (n = 3) with the corresponding heterozygous and littermate controls. As a positive control we included liver of 4-month old superoxide dismutase-deficient (*Sod1* ^−/−^) mice, which we have previously shown to contain an increased number of apoptotic cells compared to wt control mouse liver [18]. To assure reproducibility, immunohistochemistry was done using tissue arrays with positive and negative controls on the same arrays. Very few apoptotic cells were observed, either in the livers of the *Ku80* ^−/−^ mice or the heterozygous or wt controls (Fig. 3A). By contrast, a substantial number of cells bearing fragmented DNA were detected in the liver of *Sod1* ^−/−^ mice as compared to *Sod1* ^+/+^ mice (Fig. 3B), as reported previously [18]. Hence, the *Ku80* defect is not associated with enhanced apoptosis in the liver.

**Figure 4:**
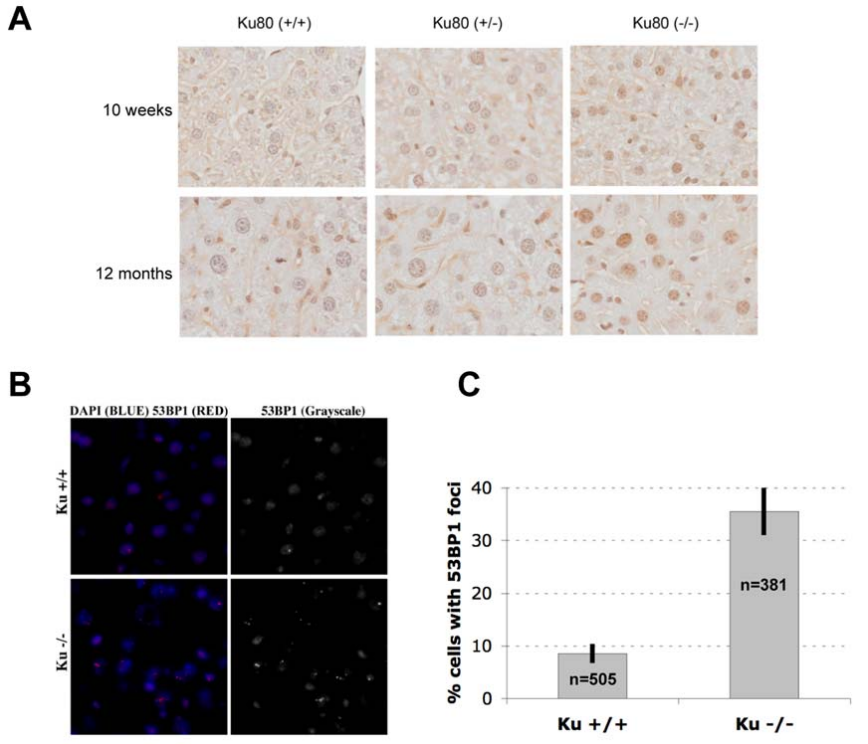
*Ku80* ^−/−^ animals accumulate markers of DNA double-strand breaks in liver. Livers from *Ku80* ^−/−^ or wt animals were analyzed using immunohistochemistry on tissue arrays (γH2AX) or immunofluorescence on fresh frozen sections (53BP1). (A) Representative γH2AX staining in livers of 10-week and 12-month old wt and *Ku80* ^−/−^ animals. (B) Representative 53BP1 staining in livers of 6-month old wt and *Ku80* ^−/−^ animals. Frozen sections were deposited on glass slides, fixed and analyzed by immunofluorescence for the presence of 53BP1 DNA damage foci. The left panels show the nuclei stained in blue (DAPI) and 53BP1 DNA damage foci (red). The right panels display the 53BP1 staining only (Grayscale). Top panels show a section from a wt liver and the lower panels show a section from a *Ku80* ^−/−^ liver. (C) The number of cells with at least one 53BP1 focus was quantified in 3 independent images from the livers of one representative pair of animals. The data are plotted as mean percentage of cells +/−S.D. from 3 independent measurements (n = total number of nuclei counted in all 3 measurements). Unpaired Student T-Test, p<0.0001 (n = 505 nuclei for wt and 381 nuclei for *Ku80* mutants).

We then examined liver tissue of *Ku80*-deficient mice for increased staining for γH2AX, a generally adopted marker for DNA double-strand breaks (DSBs) and cellular senescence [19]. Mouse embryonic fibroblasts (MEFs) from *Ku80* ^−/−^ mice undergo rapid senescence in culture and, compared to wt MEFs, more cells harbor one or more chromosomal aberration [20], [21]. Cellular senescence can be induced by agents that induce genome rearrangements, including ionizing radiation and hydrogen peroxide [22]. Under these conditions, senescent cells appear to harbor unrepaired DNA double strand breaks (DSBs), which can be visualized by immunostaining for nuclear foci containing phosphorylated (γ) H2AX or 53BP1 [23]. We therefore tested liver, an organ that showed increased genome rearrangements upon *Ku80* deficiency (Fig. 2), of wt and *Ku80* ^−/−^ mice for the presence of γH2AX using immunohistochemistry (the same tissue arrays as in the apoptosis experiments). The results clearly show that cells in the *Ku80* ^−/−^ liver almost uniformly stained positive for γH2AX (Fig. 4A). This was confirmed by independent (in three pairs of mice) immunofluorescent detection of 53BP1 foci in *Ku80* ^−/−^ as compared to wt liver (Fig. 4B and C). Hence, these results point towards an excess of unresolved DSBs in *Ku80* ^−/−^ liver, suggesting a greater number of senescent cells.

### Mutations in Cultured Fibroblasts

To better understand how *Ku80* deficiency affected mutation induction on the short term, we treated wt and *Ku80* ^−/−^ MEFs with hydrogen peroxide (H_2_O_2_), a known clastogen, which we have previously shown to induce mostly genome rearrangements [24]. We measured mutant frequencies 48 hours after exposure. We used H_2_O_2_ at a dose of 0.1 mM, which did not result in a significant loss of viability during the course of the experiment (survival >90%). *Ku80* MEFs proliferated more slowly than wt cells, despite being routinely cultured in 3% oxygen, as reported [22]. We measured lacZ mutations induced by H_2_O_2_ in both proliferating and quiescent cells. Only proliferating cells can repair DSBs by homologous recombination (HR), which is generally considered to be much less error-prone than NHEJ. Hence, one might expect a higher level of mutations in quiescent *Ku80* ^−/−^ than in proliferating cells.

**Figure 5:**
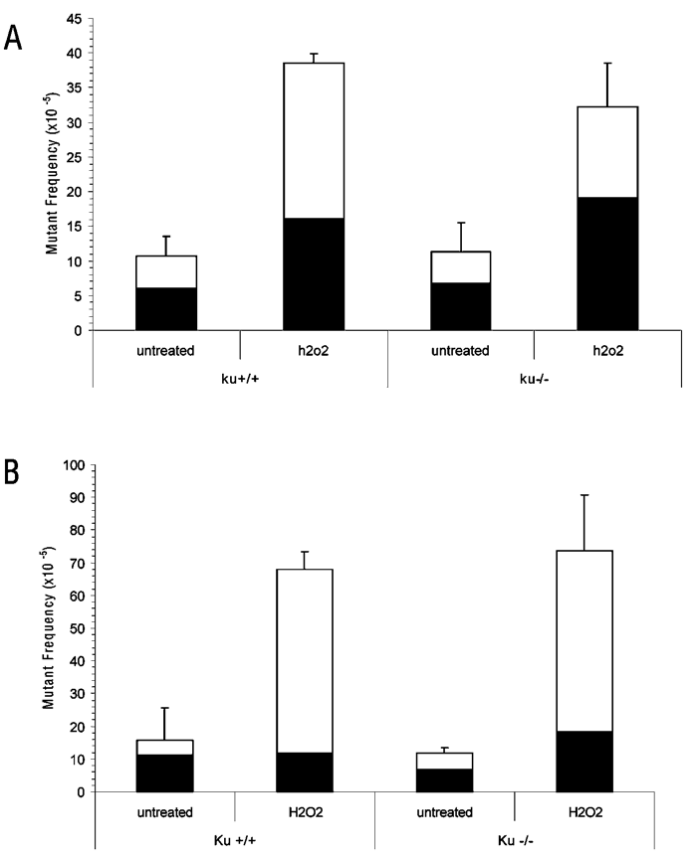
LacZ mutant frequencies in H_2_O_2_-treated *Ku80* ^−/−^ and wt MEFs. Frequencies of genome rearrangements (white) and point mutations (black) in (A) proliferating or (B) quiescent *Ku80* ^−/−^ or wt MEFs. Error bars indicate standard deviations. No statistically significant differences were observed between wt and *Ku80* ^−/−^ MEFs whether or not they were subjected to H_2_O_2_ exposure.

Surprisingly, neither proliferating nor quiescent cells showed any mutational effects of *Ku80* deficiency (Fig. 5). The increased ratio of genome rearrangements in quiescent as compared to proliferating cells was reported by us previously [24]. This is possibly due to the increased toxicity of DSBs during replication. To determine whether the genome rearrangements in proliferating cells were qualitatively different from those induced in quiescent cells–for example, because the former might result from errors during HR repair–we characterized the breakpoints of several representative rearrangements by sequencing (Table 2). As we demonstrated previously for mouse tissues, there were very few sequence homologies at these break points indicating that they arose through illegitimate recombination. No apparent differences between proliferating and quiescent *Ku80* ^−/−^ cells were observed.

**Table 2:** Breakpoints in the *lacZ* transgene and the mouse genome of genome rearrangements in proliferating and quiescent *Ku80* ^−/−^ MEFs.

Mutant ID	Sequence (5′→3′)^a^		Origin
pro_101	CGGCAGTTATCTGGAAGATCAGGATA**TGTG**	gcggatgagcggcattttccgtgacgtctc	pUR288
	atcagttatccaaaagaatttctagt**tgtg**	TCATGGTTAAGTGTGTACAACATGGTTCTT	chr. X
pro_102	GATACGCCGAACGATCGCCAGTTCTGTAT**G**	aacggtctggtctttgccgaccgcacgccg	pUR288
	ttcccttaggagtcacaaattccagggaa**g**	TACTTTTCTTGGTTTAATGTGATTGAATAA	chr. 11
pro_103	TATGTGGTGGATGAAGCCAATATTGAA**ACC**	cacggcatggtgccaatgaatcgtctgacc	pUR288
	gcccgatgtacgcgcgcgtggatgaag**acc**	AGCCCTTCCCGGCTGTGCCGAAATGGTCCA	pUR288
pro_107	GGCGGCGGAGCCGACACCACGGCCACCGAT	attatttgcccgatgtacgcgcgcgtggat	pUR288
	ataaacgatgccgactggcaatgcggcggc	GTTATTCCCATGACCCGCCGGGCAGCTTCC	chr. 17
qui_108	GTCATAGCGATAACGAGCTCCTGCACTGGA	tggtggcgctggatggtaagccgctggcaa	pUR288
	agtctcaagcctccagtgtgcgtcagttgt	ATATCTGGGGATCTCTCCACCACACAAGGG	chr. 3
qui_109	GCTGCATAAACCGACTACACAAATCAGCGA	tttccatgttgccactcgctttaatgatga	pUR288
	cctatcaaagcaaaagcaaactccactgtg	AATGTACAAATGAGCAGAAGAGCACTTGAC	chr. X
qui_111	CGTTTATCCGGGCAAACCATCGAAGTGACC	agcgaatacctgttccgtcatagcgataac	pUR288
	gggctgtggggtggcttggtgggtaatgtg	CTTGCCTCAGAAGCATGAAGATCTGCGTTG	chr. 1
qui_114	GCGCCGAAATCCCGAATCTCTATCGTGCG**G**	tggttgaactgcacaccgccgacggcacgc	pUR288
	gagaacttacttgagtgtgggtttagagt**g**	CTTTGTACATAGAGACTAACTACAGTTCTG	chr. 16

a The capitalized nucleotides represent the recovered mutant sequence. Bold letters indicate direct homology between the breakpoint in the transgene and the mouse genome.

**Figure 6:**
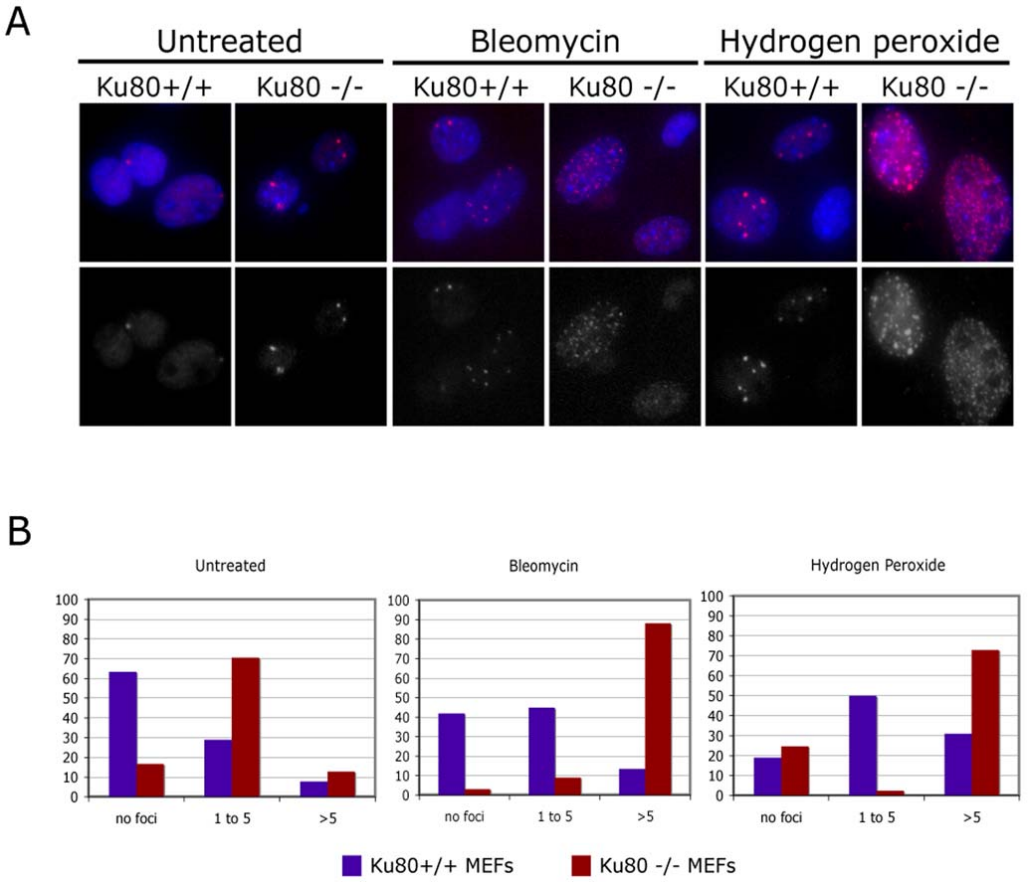
Persistent DNA damage foci in *Ku80* ^−/−^ MEFs. (A) Persistent DNA damage foci were visualized by immunofluorescence. Wt and *Ku80* ^−/−^ MEFs were treated with bleomycin or H_2_O_2_ and stained 3 d later for the presence of 53BP1 foci. Top panels–53BP1 in red and nuclei counterstained with DAPI (blue). Bottom panels–53BP1 in grayscale. (B) Quantification of DNA damage foci (53BP1) shown in A, in untreated cells and 3 d after treatment of wt and *Ku80* ^−/−^ MEFs.

To more directly determine whether H_2_O_2_-induced DSBs were as efficiently repaired in *Ku80* ^−/−^ MEFs as in wt cells, we treated proliferating wt and *Ku80* ^−/−^ cells with low doses of the clastogens H_2_O_2_ or bleomycin and then analyzed the cells for 53BP1 and γH2AX foci at 72 hours post treatment, providing ample time for repair. After this time period, residual damage, as expected, was more prominent in *Ku80* mutant cells (Fig. 6). Hence, while an approximately equal number of genome rearrangements were present after H_2_O_2_ or bleomycin treatment in *Ku80* ^−/−^ and wt cells, the number of persistent DSBs was dramatically higher in the former. Of note, the frequency of 53BP1 foci in untreated *Ku80* ^−/−^ cells was also significantly higher than that of wt cells (Fig. 6).

## Discussion

DNA damage is considered an important cause of both cancer and aging [25]. Cancer is caused by mutations, which can arise as a consequence of errors made during the repair or replication of a damaged DNA template [26]. The non-cancer related, degenerative symptoms of aging are thought to result from cellular responses to DNA damage, such as cellular senescence and apoptosis, processes that generally suppress cancer [27]. This antagonism between cancer and aging is reflected in the observation that not all heritable defects in DNA repair increase cancer risk. Several DNA repair defective mouse models, including *Ku80* ^−/−^ mice, show a reduced incidence of spontaneous tumors [5], [28]. This relative protection from cancer is likely due to the chronic activation of aggressive cellular responses to DNA damage, which are often mediated by p53 [29] and are especially critical in stem cell compartments [30]. Indeed, stem cell function is often adversely affected in DNA repair defective mice [31]. Loss of stem cell regenerative capacity is also considered an important cause of normal aging [32].

The chronically increased levels of unrepaired DSBs observed in the livers of Ku80^−/−^ mice likely continuously activate the senescence response, which in turn reduces cancer. It is now clear that senescence is as effective as apoptosis in reducing cancer incidence, albeit probably at the cost of compromising tissue regeneration and integrity [33]. In addition to persistent DSBs, which likely cause an accumulation of pro-aging senescent cells, we also found an accumulation of large genome rearrangements in *Ku80* ^−/−^ livers and spleens. These mutations likely also adversely affect organ and tissue function by deregulating gene expression [13]. Hence, the premature aging phenotype of *Ku80* deficient mice could be due to both mutation accumulation and cellular senescence, both consequences of the chronic genotoxic stress caused by loss of *Ku80* function. Cellular senescence may be responsible for the reduced number of point mutations observed in most organs of *Ku80* ^−/−^ mice. Point mutations increase the risk of developing cancer. For example, in mice lacking the mismatch repair gene *Pms2*, spontaneous mutation frequencies are elevated 100-fold in all tissues examined, with most of these mutations being small deletions and insertions [34]. Interestingly, while these mice have a very high incidence of cancer they show no signs of premature aging [35].

The reduced frequency of point mutations, which are replication-dependent [36], is likely a consequence of the lower growth rate of *Ku80* ^−/−^ cells. The slow growth rate of these cells, in turn, is likely due to the chronically high levels of DNA DSBs, which must be repaired during S phase by homologous recombination repair. Hence, it is possible that the reduced proliferation rate of *Ku80* ^−/−^ cells limits the rate of replication errors. NHEJ is expected to affect the frequency of genome rearrangements, but not the frequency of point mutations. Consistent with the idea that *Ku80* deficiency suppresses point mutations indirectly by retarding cell proliferation, we found that the reduced level of point mutations in Ku80^−/−^ cells cannot be explained by a loss of NHEJ-related small deletions (Table 1).

While a significant increase of genome rearrangements was observed in tissues of adult animals there was essentially no effect of *Ku80* deficiency on the short term, i.e., 48 h after exposure to a clastogen, neither in proliferating nor quiescent cells. Indeed, the damage induced by these agents remains largely unrepaired, as indicated by the persistency of DNA damage foci in *Ku80* ^−/−^ as compared to normal cells. These results indicate that the role of NHEJ is not immediately taken over by alternative, more error-prone pathways. Instead, the unrepaired DNA damage likely induces cellular senescence, which essentially prevents the fixation of DSBs into stable rearrangements. However, in the longer term genome rearrangements do accumulate as indicated by our observations in tissues of young, adult animals. Hence, a small fraction of the unrepaired damage is processed by alternative pathways, occasionally generating genome rearrangements.

Our observation that in the absence of clastogens there is no increase in genome rearrangements in *Ku80* ^−/−^ cells is in contrast to the reported increase in chromosomal aberrations in *Ku80^−^* ^/−^ MEFs [20]. There are two possible explanations for this discrepancy, which are not mutually exclusive. First, much of the observed chromosomal instability, for example, at telomeres [6] or aneuploidy [20] are not detected by the lacZ reporter. Second, we previously reported that normal MEFs cultured in 20% oxygen accumulate point mutations, but not rearrangements, despite the fact that the level of spontaneous rearrangements is significantly higher in MEFs compared to tissues [37]. It is possible that a growth disadvantage prevents cells from accumulating high levels of genome rearrangements, which are likely to compromise cell function more severely than point mutations. In the quiescent cells it is possible that after extended periods of time some of the persistent DSBs are eventually fixed into stable rearrangements, as is evidently the case in liver and spleen.

## Materials and Methods

### Mice

*Ku80* ^+/−^ mice on a C57/BL/6J background were crossed with C57BL/6J pUR288-(lacZ)-transgenic mice line 60 (integration sites on chromosomes 3 and 4) and bred to generate *Ku80* ^−/−^ animals hemizygous for pUR288 (lacZ). *Ku80* ^+/+^ littermate animals served as wt controls. *Ku80* ^+/−^/lacZ^+/−^ mice were retained for preparing MEFs (see below). The animals were maintained in the animal facilities of the University of Texas Health Science Center at San Antonio on a 12-hour light/12-hour dark cycle at a standard temperature of 23°C. Standard lab chow (Harlan Teklad, Madison WI) and water were supplied *ad libitum*. Animals were sacrificed by CO_2_ inhalation followed by cervical dislocation at 4–5 months of age. Ethical approval to carry out this work on animals was provided by the IACUC of the University of Texas Health Science Center.

### Cell isolation and culture

MEFs were isolated from day 13.5 embryos generated by crossing the aforementioned *Ku80* ^+/−^/lacZ^+/−^ animals among each other. MEF isolation has been described previously [37]. Briefly, the placental membranes, amniotic sac, head and primordial blood organs were removed and the remaining carcass was rinsed with phosphate buffered saline (PBS) and minced in 2 ml PBS. The tissue fragments were passed through a 18 G needle and a 100-µm strainer to remove large fragments, and placed in a 25-cm^2^ flask containing DMEM (GIBCO), 10% (v/v) fetal bovine serum (FBS; GIBCO), 50 units/ml penicillin and 50 µg/ml streptomycin. At this and subsequent stages of culture, the cells were maintained in 3% oxygen. At confluency, the cells were transferred to a 75-cm^2^ flask, cultured until 90% confluent, and then transferred to 150-cm^2^ flasks. These cells were considered passage 1 and population doubling (PD) 0. Experiments were conducted on cells at passage 3.

### Generation of quiescent cells

MEFs were prepared from individual embryos. Cells, which were either *Ku80* ^−/−^ or ^+/+^ (and lacZ ^+/−^), were plated in 10-cm dishes (10^6^ cells) in the presence of 10% FBS and penicillin/streptomycin. To induce quiescence, the cells were plated and 24 h later washed 3 times with PBS and provided with DMEM containing 0.5% serum and 1% penicillin/streptomycin. The cells were maintained in this medium throughout the period of the experiment and were used after 5 days. Visual inspection indicated no increase in cell number, which was confirmed by [3H]-thymidine incorporation experiments and FACS analysis [36].

### Treatment with hydrogen peroxide or bleomycin

Cells were washed twice with PBS and then incubated with 10 µg/ml bleomycin or 0.1 mM H_2_O_2_ (Sigma-Aldrich Inc.) in medium without serum for 2 h at 37°C. After the treatment period the cells were washed twice with PBS. For quiescent cells, the original incubation medium was retained and replaced following the treatment. Proliferating cells were provided with fresh, complete medium after exposure. Control cells were mock treated. All cells were returned to the incubator and harvested 48 h after treatment. Data obtained for proliferating cells were the result of 3 independent experiments whilst those for quiescent cells were from 2 independent experiments.

### Plasmid rescue and mutation analysis

DNA was extracted by routine phenol/chloroform extraction. Complete protocols for plasmid rescue and mutant frequency determinations with this model have been described [15]. Briefly, between 20 and 30 µg genomic DNA was digested with Hind III (Roche) for one hour in the presence of magnetic beads (Dynal) pre-coated with lacZ-lacI fusion protein. The beads were washed three times to remove excess genomic DNA. Plasmids were subsequently eluted from the beads by IPTG. After circularization of the plasmids with T4 DNA ligase, they were ethanol precipitated and used to transform Escherichia coli C (ΔlacZ, galE^−^) cells. One thousandth of the transformed cells was plated on a titer plate containing X-gal and the remainder on a selective plate containing p-gal. The plates were incubated for 15 h at 37°C. Mutant frequencies were determined from the number of colonies on the selective plate divided by the number of colonies on the titer plate (times the dilution factor of 1,000). Each mutant frequency is based on at least 300,000 recovered plasmids.

### Mutant classification and characterization

Mutant colonies were taken from the selective plates and grown at 37°C overnight in 96-well round-bottomed plates containing LB medium, kanamycin and ampicillin. One µl was then directly plated on X-gal to screen for galactose-insensitive host cells and this background was subtracted. One µl was added to a PCR mix and the DNA amplified as described [15]. The PCR products were digested with AvaI and size separated on a 1% agarose gel. No-change mutations, with restriction patterns resembling the wt pattern, consist of base changes as well as small insertions and deletions up to 50kb in size. Size-change mutations are those that deviate from the wt restriction pattern and represent rearrangements with one breakpoint in the lacZ gene and the other elsewhere in the mouse genome. Approximately 48 mutants per condition were analyzed. LacZ genes of selected mutant plasmids were prepared for sequencing. Sequencing of purified mutant plasmids was outsourced to the UC Davis sequencing facility (Davis, CA). The chromatograms were analyzed with Sequencher (Gene Codes, Ann Arbor, MI). The primers used for the sequence reactions have been described previously [15].

### Tissue arrays and immunohistochemistry

Livers were removed, formalin-fixed, paraffin-embedded, and sectioned. Tissue arrays were constructed as described previously [38]. Briefly, a H&E-stained tissue section was made from each donor block to define representative regions. Two core tissue biopsies were taken from individual paraffin-embedded liver samples (donor blocks) and arranged in a new recipient paraffin block (tissue array block) using a trephine apparatus (Superbiochips Laboratories, Seoul, Korea). Each tissue array block contained up to 60 samples. Sections of 4 µm were cut from each tissue array block, deparaffinized and dehydrated. After rinsing twice with PBS, the sections were incubated with 50 ml TUNEL reaction mixture for 1 h at 37°C, 50 ml Converter-POD for 30 min at 37°C, and 100 ml DAB-substrate solution for 2 min at room temperature. The sections were rinsed three times with PBS between each step. The slide was analyzed under a light microscope. Immunohistochemical detection of γH2AX was carried out using a mouse monoclonal antibody (05–636, Upstate Charlottesville, VA) after blocking with mouse immunoglobulin blocking reagent supplied in a MOM (mouse on mouse) detection kit (Vecter, Burlingame, CA). Biotinylated anti-mouse Ig and avidin-biotin complex were subsequently applied according to the manufacturer's instruction. Slides were treated with diaminobenzidine and counterstained with Meyer's hematoxylin.

### Immunofluorescence

Immunostaining was done on frozen sections of mouse liver (53BP1) and MEFs (both γH2AX and 53BP1). For frozen sections, three 6-month-old mice were sacrificed and livers were dipped in OCT (Tissue-Tek #4583), flash frozen in liquid nitrogen, and preserved at −80°C. Livers were sectioned at −20°C (thickness 6 µm) and slices were placed on superfrost plus microslides (VWR #48311-703). Slices were air dried and stored at −80°C until immunostaining was performed. The samples were permeabilized in PBS-0.5% Triton for 10 min. Slides were blocked for 1 h in PBS containing 1% BSA and 4% normal donkey serum. Primary antibodies were diluted in blocking buffer and incubated overnight at 4°C. The next day slides were incubated with secondary donkey antibodies for 1 h at room temperature. Finally, slides were mounted with slow-fade gold (Molecular probes) before analysis. Images were acquired on an Olympus BX60 upright fluorescence microscope with the spotfire 3.2.4 software (Diagnostics instruments) and further analyzed with Photoshop CS2 (Adobe).

MEFs from wt and *Ku80* ^−/−^ mice were plated at a density of 50,000 cells per well in a 4-well chamber slide. The next day, the cells were treated with 10 µg/ml bleomycin or 0.1 mM H_2_O_2_ for 2 h, washed with PBS and incubated in fresh medium for 3 d. Cells were then washed with PBS, fixed with 4% paraformaldehyde for 10 min at room temperature, permeabilized with 0.5% Triton X-100 for 10 min, blocked for 30 min and incubated overnight at 4°C with primary antibodies against 53BP1 or γ-H2AX. The next day, cells were washed 3 times with PBS, incubated with secondary antibodies for 45 min at room temperature, then washed again with PBS. DAPI was added in the second wash. The cells were photographed using a Nikon Eclipse 800 microscope with 40× objective and a Nikon Digital Camera DXM1200.

The primary antibody for γH2AX was a mouse monoclonal from Upstate, and the secondary antibody was goat anti-mouse IgG conjugated to Alexa 488 (Molecular Probes). The primary antibody for 53BP1 was a rabbit polyclonal antibody from Bethyl (BL182) and the secondary antibody was a donkey anti-rabbit antibody conjugated to Alexa fluor 594 (Molecular Probes). DAPI was included in the secondary antibody mixture to label nuclear DNA.

Only prominent 53BP1 foci, reminiscent of irradiation-induced foci (IRIF), were scored. All cells with one or more IRIF-like 53BP1 foci were considered positive for the presence of DSB-associated DNA damage foci.

### Statistical analysis

Unpaired t-test was used for all statistical analyses using the statistical program JMP (SAS Institutes, Inc. Cary, NC). P<0.05 was considered significant.
